# Effective Three-Stage Demosaicking Method for RGBW CFA Images Using The Iterative Error-Compensation Based Approach

**DOI:** 10.3390/s20143908

**Published:** 2020-07-14

**Authors:** Kuo-Liang Chung, Tzu-Hsien Chan, Szu-Ni Chen

**Affiliations:** Department of Computer Science and Information Engineering, National Taiwan University of Science and Technology, Taipei 10672, Taiwan; M10715077@mail.ntust.edu.tw (T.-H.C.); M10815013@mail.ntust.edu.tw (S.-N.C.)

**Keywords:** color difference, demosaicking, demosaiced RGB full-color image, error compensation, perceptual effect, quality, RGBW color filter array (CFA) image

## Abstract

As the color filter array (CFA)2.0, the RGBW CFA pattern, in which each CFA pixel contains only one R, G, B, or W color value, provides more luminance information than the Bayer CFA pattern. Demosaicking RGBW CFA images $I^{RGBW}$ is necessary in order to provide high-quality RGB full-color images as the target images for human perception. In this letter, we propose a three-stage demosaicking method for $I^{RGBW}$. In the first-stage, a cross shape-based color difference approach is proposed in order to interpolate the missing *W* color pixels in the *W* color plane of $I^{RGBW}$. In the second stage, an iterative error compensation-based demosaicking process is proposed to improve the quality of the demosaiced RGB full-color image. In the third stage, taking the input image $I^{RGBW}$ as the ground truth RGBW CFA image, an $I^{RGBW}$-based refinement process is proposed to refine the quality of the demosaiced image obtained by the second stage. Based on the testing RGBW images that were collected from the Kodak and IMAX datasets, the comprehensive experimental results illustrated that the proposed three-stage demosaicking method achieves substantial quality and perceptual effect improvement relative to the previous method by Hamilton and Compton and the two state-of-the-art methods, Kwan *et al.*’s pansharpening-based method, and Kwan and Chou’s deep learning-based method.

## 1. Introduction

Most modern digital color cameras are equipped with a single sensor covered with a Bayer color filter array (CFA) pattern [1] such that each Bayer CFA pixel contains only one red (R), green (G), or blue (B) color value. The captured Bayer CFA image $I^{Bayer}$ consists of 25% R, 50% G, and 25% B color values. Besides the low hardware cost advantage, $I^{Bayer}$ can usually be demosaicked to a high-quality RGB full-color image while using the demosaicking method in $I^{Bayer}$ [2,3,4,5,6,7]. However, in the low illumination condition, the thermal noise side-effect of $I^{Bayer}$ leads to low quality of the reconstructed RGB full-color image [8]. Therefore, to receive more luminance than that of the Bayer CFA pattern, a single sensor covered with a RGBW CFA pattern [9], in which one pixel contains only one R, G, B, or white (W) color value, has been incorporated into some digital color cameras and mobile phones, such as the Huawei-Ascend series, Huawei G8, Huawei P8, Huawei Mate S, and Oppo R7 Plus. The captured image by such a device is called the RGBW CFA image $I^{RGBW}$.

**Figure 1:**
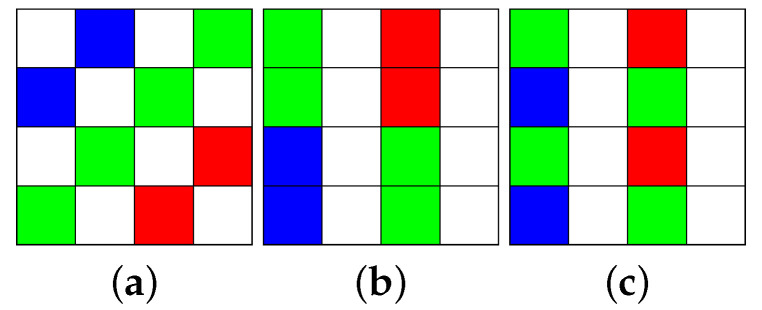
The three commonly used RGBW-Kodak CFA patterns. (**a**) RGBW-Kodak-1. (**b**) RGBW-Kodak-2. (**c**) RGBW-Kodak-3.

The three commonly used 4×4 RGBW-Kodak CFA patterns [10] are depicted in Figure 1a–c. From practical experience, Compton and Hamilton [10] found that the *W* color photoresponse is three to four times more sensitive to wide spectrum light than R-, G-, or B-color photoresponse. To balance the same sensitivity as the W pixels, they suggested increasing the size of the R, G, and B color pixels. This is the reason why the RGBW CFA patterns were proposed. For convenience, we take the RGBW-Kodak-1 CFA pattern as the representative pattern in our discussion, although our discussion is also applicable to the other two RGBW-Kodak CFA patterns.

Before providing a RGB full-color image as the target image for human perception, demosaicking the RGBW CFA image $I^{RGBW}$ is a necessary step. Because the RGBW CFA patterns are different from Bayer CFA patterns, the existing demosaicking methods for $I^{Bayer}$ cannot be directly used to demosaic $I^{RGBW}$. In what follows, we introduce the related demosaicking methods for $I^{RGBW}$.

### 1.1. Related Work

Given an $I^{RGBW}$, Hamilton and Compton [11] first partition each 4 × 4 RGBW CFA block (see Figure 1a) into two disjointed 4 × 4 CFA blocks in which one contains only eight *W* color values, called the W-CFA block, and the other contains two *R* color values, four *G* color values, and two *B* color values, called the RGB-CFA block. For the whole RGB-CFA image, they average the two same color values to produce a quarter-sized Bayer CFA image $I^{q,Bayer}$. Next, $I^{q,Bayer}$ is demosaicked to a RGB full-color image $I^{q,RGB}$. For the whole W-CFA image, first, a bilinear interpolation is applied to recover all the missing *W* color values for producing a *W* color image $I^{W}$. Then, they apply an averaging-based downsampling on $I^{W}$ to produce a quarter-sized *W* image $I^{q,W}$. Furthermore, a bilinear interpolation is applied to upsample the three quarter-sized images, producing the three color difference images, $I^{R-W}$, $I^{G-W}$, and $I^{B-W}$. Finally, adding $I^{W}$ to $I^{R-W}$, $I^{G-W}$, and $I^{B-W}$, respectively, produces the required R, G, and B color planes as the demosaiced RGB full-color image $I^{RGB}$.

Condat [12] proposed a variational approach to demosaick $I^{RGBW}$ (see Figure 1. (III) in [12]), which is exactly the RGBW-Kodak-1 in Figure 1, such that the demosaiced RGB full-color image has maximal smoothness under the constraint of consistency measurements. For convenience, his variational approach method is called the VA method. In VA, Condat considered the orthonormal basis corresponding to luminance, red-green, and blue-yellow chrominances. Furthermore, he transformed the problem of demosaicking $I^{RGBW}$ into a minimization problem with regularization function. Finally, an iterative numerical process was delivered to solve the problem.

Kwan et al. [13] observed that the downsampling process on the upsampled W-CFA image $I^{W}$ may lose useful information in $I^{W}$. To recover as much as the lost information in $I^{W}$ as possible, they employed a hybrid color mapping-based pansharpening technique [14] in Hamilton and Compton’s method in order to reconstruct a better demosaiced RGB full-color image. First, they demosaicked $I^{q,Bayer}$ to obtain $I^{q,RGB}$ using Zhang et al.’s method [7], and then they stacked the R and G planes of $I^{q,RGB}$ to $I^{q,W}$, obtaining $I^{q,RGW}$. Secondly, by solving a minimization problem with a regularization term, they derived a matrix *T* to transform $I^{q,RGW}$ to $I^{q,RGB}$. Thirdly, they stacked the upsampled version of $I^{q,R}$ and $I^{q,G}$ to $I^{W}$, obtaining $I^{RGW}$. Finally, with the help of *T*, $I^{RGW}$ is transformed to the RGB full-color image $I^{RGB}$.

Based on the convolutional neural networks-based framework, namely ”DEMONET” [15], Kwan and Chou [16] applied the trained version of DEMONET to demosaic $I^{q,Bayer}$, which was obtained using Hamilton and Compton’s method, producing the demosaiced quarter-sized RGB full-color image $I^{q,RGB}$. Next, they created a 2×2 fictitious Bayer pattern in which the *W* color pixel in $I^{W}$ is treated as the *G* color pixel;the *R* color pixel and the *B* color pixel come from the $I^{q,RGB}$. Furthermore, they applied DEMONET to the fictitious Bayer CFA image again, producing a RBW full-color image $I_{DEMONET}^{RBW}$. Furthermore, they extracted the W color image $I_{DEMONET}^{W}$ from $I_{DEMONET}^{RBW}$. With the help of the same matrix T [13], they performed the pansharpening technique [14] on $I^{q,RGB}$ and $I_{DEMONET}^{W}$ twice, and then the demosaiced RGB full-color image is obtained.

Based on new inter-pixel chrominance capture and optimal demosaicking transformation, Zhang et al. [17] proposed an effective universal demosaicking method, and the experimental results demonstrated the significant quality superiority of the demosaiced images for six kinds of CFA images, including the RGBW CFA pattern in Figure 1a. In [18], Amba et al. pointed out the time consuming problem in [17].

Among the above-mentioned six related methods, when considering the available codes, the former four methods are included in the comparative methods. For convenience, the methods by Hamilton and Compton [11], Kwan et al. [13], and Kwan and Chou [16] are called the HC method, the pansharpening-based method, and the deep learning-based method, respectively.

### 1.2. Contributions

For $I^{RGBW}$, this letter proposes an effective three-stage demosaicking method. In the first stage, the proposed cross shape-based color difference technique is used to reconstruct the missing *W* color pixels in the *W* color plane of $I^{RGBW}$ more effectively, and the reconstructed *W* color plane has a good edge-preserving effect. In the second stage, based on the interpolated *W* color pixels and $I^{RGBW}$, an iterative error compensation-based demosaicking process is proposed in order to reduce the demosaiced error in the *R*, *G*, and *B* color planes of the demosaiced RGB full-color image. In the third stage, taking $I^{RGBW}$ as the ground truth RGBW CFA image, the $I^{RGBW}$-based refinement approach is proposed to improve the result obtained by the second stage, achieving a better demosaiced RGB full-color image.

Based on the testing RGBW CFA images collected from the Kodak and IMAX datasets, the comprehensive experimental results demonstrated that our three-stage demosaicking method achieves substantial quality improvement of the demosaiced RGB full-color images when compared with the HC method [11], the VA method [12], the pansharpening-based method [13], and the deep learning-based method [16]. In addition, the perceptual effect merit of our three-stage method is illustrated relative to the four comparative methods.

The rest of this letter is organized, as follows. In Section 2, for $I^{RGBW}$, the proposed three-stage demosaicking method is presented. In Section 3, the comprehensive experiments are carried out to demonstrate the quality and perceptual effect merits of our three-stage method. In Section 4, some concluding remarks are addressed.

## 2. The Proposed Three-Stage Demosaicking Method For RGBW CFA Images

The proposed demosaicking method for $I^{RGBW}$ consists of three new stages: (1) the cross shape-based color difference approach to reconstruct the missing *W* color pixels in the *W* color plane of $I^{RGBW}$, (2) the iterative error compensation process to minimize the error in the R, G, and B color planes of the demosaiced RGB full-color image, and (3) the $I^{RGBW}$-based refinement approach to improve the result that was obtained by the second stage, achieving a better demosaiced RGB full-color image.

### 2.1. The First Stage: The Cross Shape-Based Color Difference Approach To Construct The Missing *W* Color Pixels

**Figure 2:**
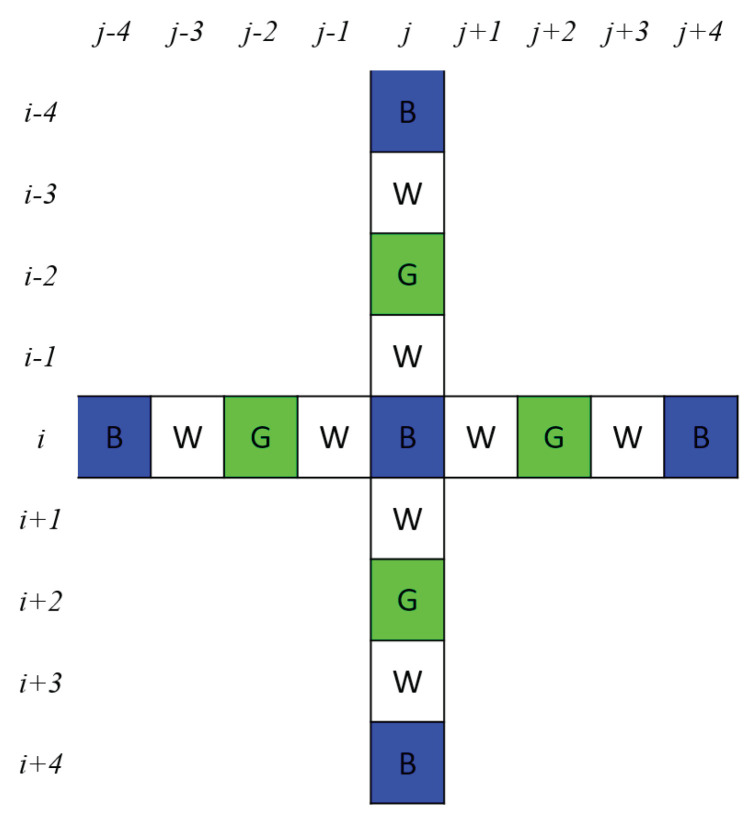
The mask used in our cross shape-based color difference approach to reconstruct the missing *W* color pixels in the *W* color plane of $I^{RGBW}$.

In this stage, we propose a simple and fast cross shape-based color difference approach to reconstruct the missing *W* color pixels in the *W* color plane of $I^{RGBW}$. First, we put a cross shape centered at the B color pixel in Figure 1a, as shown in Figure 2. We only discuss how to reconstruct the *W* color value in the *B* color pixel $I_{B}^{RGBW}$, but our discussion is also applicable to reconstruct *W* color values in the *R* and *G* color pixels. Besides the above regular cross shaped color difference approach, when considering the other B pixels, which lie off the cross, in the 9 × 9 window may improve the estimation of the W color value, although it takes more time. In the same way, our discussion is also applicable to the other two RGBW-Kodak CFA patterns presented in Figure 1b,c, and we just apply the X shaped color difference approach by considering the diagonal pixel positions.

In the proposed cross shape-based color difference approach, to reconstruct the *W* color value in the *B* color pixel $I_{B}^{RGBW}\left(i,j\right)$, as shown in Figure 2, we consider the four neighboring *W* color pixels of $I_{B}^{RGBW}\left(i,j\right)$, which are located in the set $W^{W}\left(i,j\right)$ = {(i±1,j),(i,j±1)}. Simultaneously, we also consider the four neighboring *B* color pixels of $I_{B}^{RGBW}\left(i,j\right)$ and the four neighboring *B* color pixels are located in the set $W^{B}\left(i,j\right)$ = {(i±4,j),(i,j±4)}. Based on our cross shape-based color difference approach, the reconstructed *W* color value in $I_{B}^{RGBW}\left(i,j\right)$, denoted by $I^{W}\left(i,j\right)$, can be calculated by

(1) $$\begin{array}{c} I^{W}\left(i,j\right)=I_{B}^{RGBW}\left(i,j\right)+\frac{\sum_{\left(x,y\right)\in W^{W}\left(i,j\right)}\omega_{D_{\mathrm{WB}}}\left(x,y\right)D_{\mathrm{WB}}\left(x,y\right)}{\sum_{\left(x,y\right)\in W^{W}\left(i,j\right)}\omega_{D_{\mathrm{WB}}}\left(x,y\right)} \end{array}$$

where $D_{\mathrm{WB}}\left(x,y\right)$ denotes the color difference value at location (x,y). To reconstruct $I^{W}\left(i,j\right)$, we must consider four such color difference values. For simplicity, we only explain how to calculate the color difference value at location (i+1,j), but it is applicable to the other three locations. The color difference value $D_{WB}\left(i+1,j\right)$ is calculated by

(2) $$D_{\mathrm{WB}}\left(i+1,j\right)=I_{W}^{RGBW}\left(i+1,j\right)-I_{B}^{RGBW}\left(i+4,j\right)$$

The $\omega_{D_{\mathrm{WB}}}\left(x,y\right)$ denotes the weight of $D_{\mathrm{WB}}\left(x,y\right)$, and it can be calculated by

(3) $$\begin{array}{c} \omega_{D_{\mathrm{WB}}}\left(x,y\right)={\left(\begin{array}{c} 1+\sum_{\left(p,q\right)\in W^{W}\left(i,j\right)}\left|D_{\mathrm{WB}}\left(x,y\right)-D_{\mathrm{WB}}\left(p,q\right)\right| \end{array}\right)}^{-1} \end{array}$$

Furthermore, we apply the fusion strategy to achieve better reconstructed *W* color values. If the standard deviation of the four neighboring *W* color pixels in the domain $W^{W}\left(i,j\right)$ is larger than or equal to the threshold *T*, empirically *T* = 10, Equation (1) is adopted to reconstruct the *W* color value at location (i,j), i.e., $I^{W}\left(i,j\right)$; otherwise, the refined value of $I^{W}\left(i,j\right)$ is fused by

(4) $$I^{W}\left(i,j\right)=\alpha \times W_{mean}+\left(1-\alpha \right)\times I^{W}\left(i,j\right)$$

where $W_{mean}$ denotes the mean value of the four *W* color values in the domain $W^{W}\left(i,j\right)$; the value of $I^{W}\left(i,j\right)$ at the right hand side of Equation (4) is obtained by Equation (1); empirically, the best choice of α is set to 0.6 after examining all possible values from 0.1, 0.2, ..., 0.5, 0.6, ..., 0.8, and 0.9.

### 2.2. The Second Stage: The Iterative Error Compensation Approach To Minimize The R, G, B Errors

This stage consists of two steps. In the first step, we modify the HC method [11] to produce a rough demosaiced RGB full-color image $I^{rough,RGB}$. In the second step, we propose a novel iterative error compensation-based approach in order to substantially enhance the quality of $I^{rough,RGB}$.

*(1) Producing the rough demosaiced image $I^{rough,RGB}$:* first, we perform an average-based downsampling process on $I^{W}$, which has been obtained by the first stage, to produce a quarter-sized *W* image, namely $I^{q,W}$. Next, we perform the averaging-based downsampling process on the RGB-CFA image, as mentioned in the first paragraph of Section 1.1, to obtain the quarter-sized Bayer CFA image $I^{q,Bayer}$. Subsequently, $I^{q,Bayer}$ is demosaicked to a full-color image $I^{q,RGB}$, and $I^{q,RGB}$ is decomposed into three color images, namely $I^{q,R}$, $I^{q,G}$, and $I^{q,B}$.

Furthermore, three quarter-sized color difference images, $I^{q,R-W}$ (= $I^{q,R}$−$I^{q,W}$), $I^{q,G-W}$ (= $I^{q,G}$−$I^{q,W}$), and $I^{q,B-W}$ (= $I^{q,B}$−$I^{q,W}$), are created, and then a bilinear-based upsampling process is performed on the three quarter-sized color difference images. As a result, it yields the three color difference images, $I^{R-W}$, $I^{G-W}$, and $I^{B-W}$. Finally, we add $I^{W}$ back to $I^{R-W}$, $I^{G-W}$, and $I^{B-W}$, respectively, for producing the three color images, $I^{R}$, $I^{G}$, and $I^{B}$; after combining the three color images, the rough demosaiced RGB full-color image $I^{rough,RGB}$ is followed. Due to applying our cross shape-based color difference approach in order to reconstruct a better *W* color plane $I^{W}$, $I^{rough,RGB}$ has better quality than that of Hamilton and Compton’s method [11].

However, as described in the next subsection, we point out the distortion between the input RGBW CFA image $I^{RGBW}$ and the distorted RGBW CFA image, denoted by $I_{distorted}^{RGBW}$, which is extracted from $I^{rough,RGB}$. The distortion in $I_{distorted}^{RGBW}$ prompts us to develop a new iterative compensation-based approach to further enhance the quality of $I^{rough,RGB}$.

*(2) Iterative error compensation-based approach to enhance the quality of the rough demosaiced image $I^{rough,RGB}$*: in this subsection, we first define the error RGBW CFA image, denoted by $I_{err}^{RGBW}$, to be equal to $I_{distorted}^{RGBW}$ - $I^{RGBW}$ and $I_{distorted}^{RGBW}$ is often somewhat different from $I^{RGBW}$. Secondly, we propose an iterative error compensation-based approach to further enhance the quality of $I^{rough,RGB}$.

**Figure 3:**
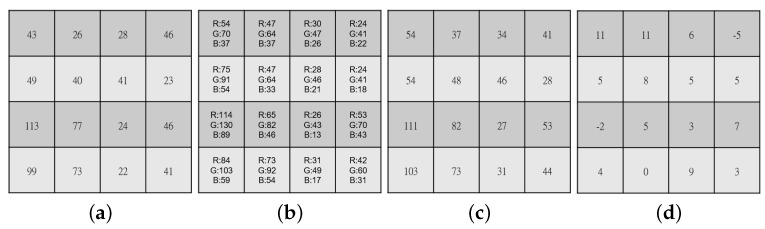
The construction of the error RGBW CFA block $B_{err}^{RGBW}$. (**a**) The original 4 × 4 RGBW CFA block $B^{RGBW}$. (**b**) The rough demosaiced block $B^{rough,RGB}$. (**c**) The distorted RGBW CFA block $B_{distorted}^{RGBW}$. (**d**) The error RGBW CFA block $B_{err}^{RGBW}$.

We take a real 4 × 4 block to explain how to construct the error RGBW CFA block $B_{err}^{RGBW}$. Figure 3a depicts the original 4 × 4 RGBW CFA block $B^{RGBW}$. Figure 3b shows the corresponding rough demosaiced RGB full-color block $B^{rough,RGB}$ by the method described in the first step of the first paragraph of Section 2.2. By the equation: W = $\frac{1}{3}$(R + G + B), the *W* color value of the top-left corner of $B^{rough,RGB}$ in Figure 3b is equal to 54 and so is the collocated *W* color value of the distorted RGBW CFA block $B_{distorted}^{RGBW}$ in Figure 3c. However, the collocated *W* color value of $B^{RGBW}$ in Figure 3a is 43. Therefore, the *W* color value of the top-left corner of $B_{err}^{RGBW}$ in Figure 3d is 11 (= 54 − 43). Similarly, the *G* color value of the top-right corner of Figure 3b is 41, but the collocated *G* color value of Figure 3a is 46. Therefore, the *G* color value of the top-right corner of the error RGBW CFA block in Figure 3d is −5 (= 41 − 46). Consequently, Figure 3d illustrates the error RGBW CFA block $B_{err}^{RGBW}$.

From the observation of the error RGBW CFA block in Figure 3d, to enhance the quality of $I^{rough,RGB}$, we propose an iterative error compensation-based approach to minimize the *R*, *G*, and *B* pixel-errors in $I^{rough,RGB}$. Similar to the demosaicking method on $I^{RGBW}$ to obtain $I^{rough,RGB}$, we first apply it to demosaic the error RGBW CFA image $I_{err}^{RGBW}$ to obtain the demosaiced error RGB full-color image $I_{err}^{RGB}$.

Subsequently, $I^{rough,RGB}$ is improved by performing the following error-compensation process:

(5) $$I^{improved,RGB}:=I^{rough,RGB}-I_{err}^{RGB}$$

**Figure 4:**
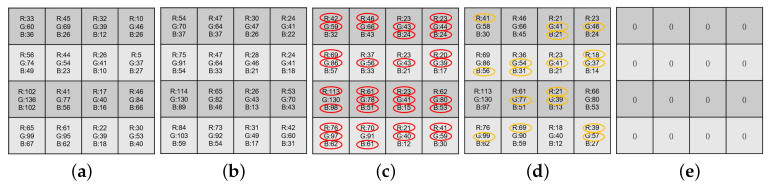
The quality improvement by the second and third stages of our three-stage demosaicking method. (**a**) The original 4 × 4 RGB full-color block $B^{RGB}$. (**b**) The rough demosaiced block $B^{rough,RGB}$. (**c**) As marked by red ellipses, the improved demosaiced R, G, and B, color values in $B^{improved,RGB}$ by our iterative error compensation-based approach. (**d**) As marked by yellow ellipses, the improved demosaiced *R*, *G*, and *B*, color values in $B^{refined,RGB}$ by our $I^{RGBW}$-based refinement process. (**e**) The error RGBW CFA block of $B^{refined,RGB}$ has been zeroized.

Based on the Kodak and IMAX datasets, “iterative number = 10” for the above error-compensation process is determined by the stop criterion which is dependent on the average absolute difference per pixel between two consecutive error RGBW maps, and when the average absolute difference per pixel is lower than the threshold value 0.5. Let the resultant demosaiced RGB full-color image be denoted by $I^{improved,RGB}$. After running our iterative error compensation-based approach on Figure 4a, as marked by red ellipses in Figure 4c, the quality of the several demosaiced *R*, *G*, and *B* color values in $B^{rough,RGB}$ by our iterative error compensation-based approach has been improved.

### 2.3. The Third Stage: The $I^{RGBW}$-Based Refinement Process To Zeroize $I_{err}^{improved,RGBW}$

In this stage, utilizing the input RGBW CFA image $I^{RGBW}$ as the ground truth RGBW CFA reference base, we propose an $I^{RGBW}$-based refinement process to further improve the quality of $I^{improved,RGB}$, which has been obtained by the second stage. The main idea in the third stage is to zeroize the error RGBW CFA image of $I^{improved,RGB}$, where $I_{err}^{improved,RGBW}$ = $I_{distorted}^{improved,RGBW}$ − $I^{RGBW}$, and $I_{distorted}^{improved,RGBW}$ is extracted from $I^{improved,RGB}$.

This stage consists of two steps. In the first step, we correct the *c* color pixel in $I^{improved,RGB}\left(x,y\right)$, c∈{R,G,B}, which is collocated with the same color pixel in $I^{RGBW}\left(x,y\right)$, and the c color is corrected by performing the assignment operation: $c:=I^{RGBW}\left(x,y\right)$. After that, the *c* color pixel in $I^{improved,RGB}\left(x,y\right),c\in \left\{R,G,B\right\}$, which is collocated with the same color pixel in $I^{RGBW}\left(x,y\right)$, has been corrected. For convenience, the refined version of $I^{modified,RGB}\left(x,y\right)$ is denoted by $I^{refined,RGB}\left(x,y\right)$.

In the second step, we refine the R, G, and B color values in $I^{improved,RGB}\left(x^{\prime },y^{\prime }\right)$, say $c_{r}$, $c_{g}$, and $c_{b}$, respectively, which are collocated with the *W* color pixel in $I^{RGBW}\left(x^{\prime },y^{\prime }\right)$. Note that the location set {$\left(x^{\prime },y^{\prime }\right)$} considered in the second step and the location set {(x,y)} considered in the first step are disjointed. The refinement operation in the second step is performed by: $I^{refined,RGB}\left(x^{\prime },y^{\prime }\right)$:= $I^{improved,RGB}\left(x^{\prime },y^{\prime }\right)$ + *k* where *k* = $W-\frac{1}{3}\left(c_{r}+c_{b}+c_{g}\right)$. After that, at location $\left(x^{\prime },y^{\prime }\right)$, from the sum of the refined R, G, and B color values being equal to 3*W*, i.e., $\left(c_{r}+c_{g}+c_{b}\right)+3W-\left(c_{r}+c_{b}+c_{g}\right)=3W$, we thus conclude that the *W* color value derived from $I^{refined,RGB}\left(x^{\prime },y^{\prime }\right)$, which is collocated with the *W* color value in $I^{RGBW}\left(x^{\prime },y^{\prime }\right)$, has been corrected.

Consequently, all of the error entries in $I_{err}^{improved,RGBW}$ have been corrected, i.e., zeroized. After performing the third stage on Figure 4d, the error RGBW CFA block of $B^{refined,RGB}$ is a 4 × 4 zero block, as shown in Figure 4e.

After describing the proposed $I^{RGBW}$-based refinement process, as marked by the yellow ellipses in Figure 4d, we observe that most color pixels in the refined 4 × 4 demosaiced RGB full-color block $B^{refined,RGB}$ are equal to or closer to those in the 4 × 4 ground truth RGB full-color block $B^{RGB}$ in Figure 4a, relative to $B^{improved,RGB}$ in Figure 4c, indicating the quality improvement merit of our $I^{RGBW}$-based refinement process by zeroizing the error RGBW image $I_{err}^{improved,RGBW}$.

## 3. Experimental Results

**Figure 5:**
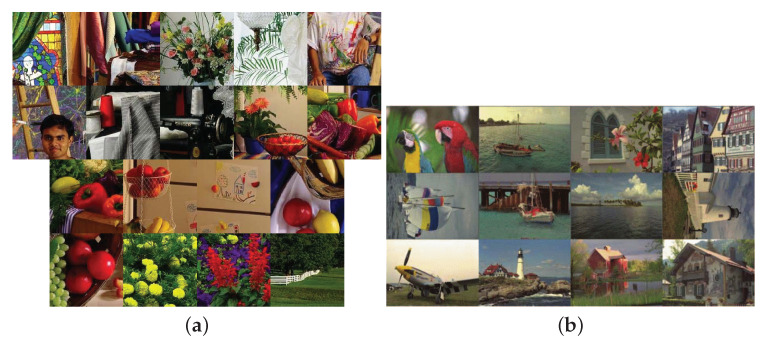
The testing IMAX and Kodak datasets. (**a**) The 18 testing images in the IMAX dataset. (**b**) The 12 testing images in the Kodak dataset.

For fairness in comparison with the two state-of-the-art demosaicking methods [13,16] without available codes, in the first set of experiments, the same 18 RGBW CFA images and 12 RGBW CFA images that were collected from the IMAX dataset [19] and the Kodak dataset [20], as shown in Figure 5a,b, respectively, are adopted to demonstrate the quality merit of our method. For completeness, in the second set of experiments, the Kodak dataset with 24 images and the IMAX dataset are used to demonstrate the quality merit of our method. In the next paragraph, the detailed implementation ways for the considered methods are described.

In the HC method, as a subroutine to convert a quarter-sized Bayer CFA image $I^{q,Bayer}$ to a quarter-sized RGB full-color image $I^{q,RGB}$, we have tried the bicubic interpolation-based demosaicking process, and this version of the HC method is called the HC${}^{bicubic}$ method. Besides the bicubic interpolation-based demosaicking process used in HC, we have also tried Kiku et al.’s demosaicking process [3] in HC, and the second version of the HC method is called the HC${}^{Kiku}$ method. The available execution codes of the HC${}^{bicubic}$ and HC${}^{Kiku}$ methods [11] can be accessed from the website [21]. Thankful for the available codes for the VA method [22], the VA method is included in the comparative methods. Since the complete codes for the pansharpening-based method [13] and the deep learning-based method [16] are unavailable, we adopt the experimental data from their papers.

Similarly, as a subroutine in our method, we have tried Kiku et al.’s method [3] and Zhang et al.’s method [7] to demosaick $I^{q,Bayer}$ to $I^{q,RGB}$, respectively. However, the average CPSNR values of our method associated with either of them are similar. The available execution code of our method can be accessed from the website [23].

All of the considered experiments are implemented on a computer with an Intel Core i7-8700 CPU 3.2 GHz and 32 GB RAM. The operating system is the Microsoft Windows 10 64- bit operating system. The program development environment is Visual C++ 2017.

### 3.1. Object Quality Merit of Our Method

Let P={(x,y)|1≤x≤H,1≤y≤W} denote the set of pixel coordinates in one RGB full-color image of size W×H. The CPSNR (color peak signal-to-noise ratio) of one demosaiced RGB full-color image is expressed as

(6) $$\mathrm{CPSNR}=10\log_{10}\frac{255^{2}}{CMSE}$$

with

(7) $$CMSE=\frac{1}{3WH}\sum_{p\in P}\sum_{c\in \{R,G,B\}}{\left[I_{n,c}^{ori,RGB}\left(p\right)-I_{n,c}^{rec,RGB}\left(p\right)\right]}^{2}$$

$I_{n,c}^{ori,RGB}\left(p\right)$ and $I_{n,c}^{rec,RGB}\left(p\right)$ denote the *c*(∈{R,G,B}) color values of the pixels at position *p* in *n*th original and the *n*th demosaiced RGB full-color images, respectively.

In the first set of experiments, for the six considered demosaicking methods, Table 1 tabulates the CPSNR values for IMAX and Kodak separately; in addition, the related average CPSNR values and the average CPSNR gains are listed. In Table 1, we observe that the proposed three-stage method, abbreviated as “Proposed”, has the best Kodak CPSNR and average CPSNR values in boldface among the considered methods. The average CPSNR gain of our method over HC${}^{bicubic}$ is 5.992 (= 35.072 − 29.08) dB. In addition, the average CPSNR gains of our method are 3.472 (= 35.072 − 31.60) dB, 1.572 (= 35.072 − 33.50) dB, 1.652 (= 35.072 − 33.42) dB, and 0.652 (= 35.072 − 34.42) dB when compared with the $HC^{Kiku}$ method, the VA method, the pansharpening-based method [13], and the deep learning-based method [16], respectively.

However, for completeness, in the second set of experiments, based on the Kodak dataset with 24 images and the IMAX dataset, in terms of CPSNR, SSIM (structural similarity index), and the average ΔE, Table 2 indicates the objective quality merit of our method relative to the other three comparative methods. Here, SSIM is measured by the joint effects of the luminance, contrast, and structure similarity preserving effect between the ground-truth RGB full-color image and the reconstructed analogue. We recommend that readers refer to [24] for a detailed definition of SSIM. The average ΔE denotes the average CIE LAB error per pixel between the ground-truth LAB image, which is converted from the ground-truth RGB full-color image, and the reconstructed analogue. We recommend that the readers refer to [17] for the definition of ΔE.

### 3.2. Perceptual Effect Merit of Our Method

Besides illustrating the CPSNR merit of our method, this subsection demonstrates the perceptual effect merit of our method relative to the comparative methods [11,12,13,16].

**Figure 6:**
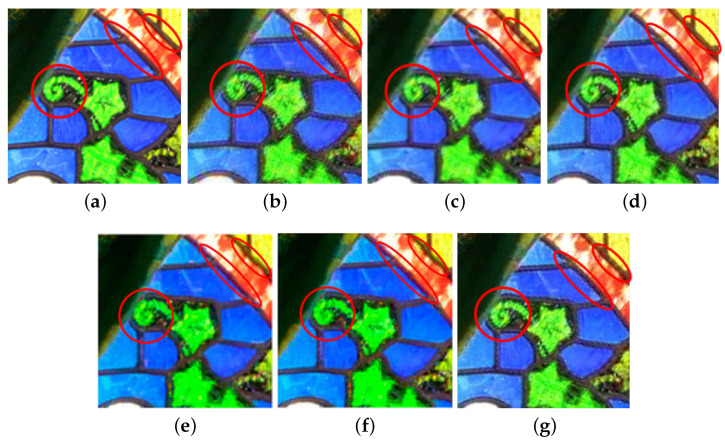
The perceptual effect merit of the proposed method for the testing image “IMAX 1. (**a**) The magnified subimage cut off from the ground truth image. (**b**) The HC${}^{bicubic}$ method [11]. (**c**) The HC${}^{Kiku}$ method [11]. (**d**) The VA method [12]. (**e**) The pansharpening-based method [13]. (**f**) The deep learning-based method [16]. (**g**) Our three-stage method.

We take the magnified subimage cut off from the testing image “IMAX 1”, as shown in Figure 6a, to show the perceptual effect merit of our method. After performing the considered demosaicking methods on the RGBW CFA image of Figure 6a, Figure 6b–g demonstrate the six demosaiced RGB full-color subimages, respectively. When compared with $HC^{bicubic}$ [11], $HC^{Kiku}$ [11], and VA [12], as highlighted by the red ellipses, our method has the best perceptual effect (also refer to the SSIM gain of our method in Table 2) and the least color shifting side effect (also refer to the average ΔE gain of our method in Table 2). As shown in Figure 6e,g, our method has better perceptual effect relative to the pansharpening-based method [19]. As shown in Figure 6f,g, the perceptual effect of our method is quite competitive to the deep learning-based method [13]; in detail, our method has better edges inside the ellipses, but our method is noisier inside the circle and the star.

**Figure 7:**
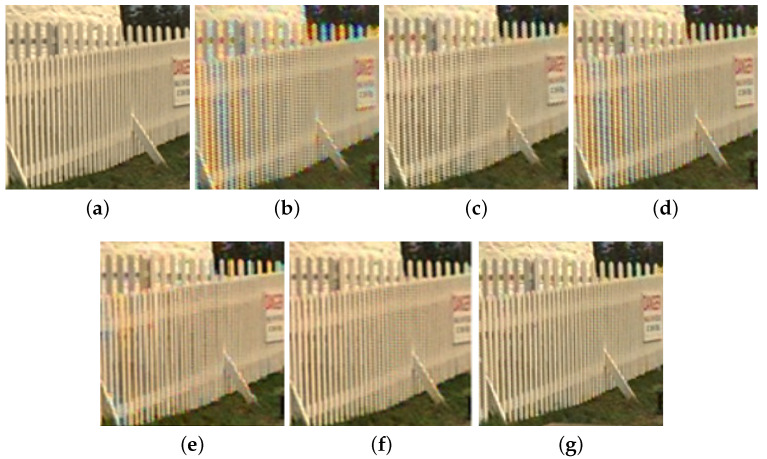
The perceptual effect merit of the proposed method for the testing image “Kodak 8”. (**a**) The magnified subimage cut off from the ground truth image. (**b**) The HC${}^{bicubic}$ method [11]. (**c**) The HC${}^{Kiku}$ method [11]. (**d**) The VA method [12]. (**e**) The pansharpening-based method [13]. (**f**) The deep learning-based method [16]. (**g**) Our three-stage method.

We also take the magnified subimage cut off from the testing image “Kodak 8”, as shown in Figure 7a, to show the perceptual effect merit of our method. After performing the considered demosaicking methods on the RGBW CFA image of Figure 7a, Figure 7b–g demonstrate the six demosaiced RGB full-color subimages, respectively. As shown in the fences, our three-stage method has the best perceptual effect and the least rainbow side effect.

### 3.3. Actual Time Cost of Our Method

In this subsection, the actual time cost of our method is reported. For $I^{RGBW}$, two different image resolutions, namely 1280 × 720 and 1440 × 1080, are created from the Kodak dataset. The image resolution 1280 × 720 is often used for HD DVD and Blu-ray DVD; 1440 × 1080 is often used for HDV. The experimental data indicated that for one 1280 × 720 image, the average execution time is 11.58 s; for one 1440 × 1080 image, the average execution time is 19.92 s. At the above-mentioned two image resolutions, the proposed method is only able to process video frames offline.

## 4. Conclusions

We have presented our three-stage method for demosaicking RGBW CFA images. In the first stage, we propose a cross shape-based color difference approach to reconstruct the missing *W* color pixels in the *W* color plane of $I^{RGBW}$. In the second stage, first, based on $I^{RGBW}$ and the reconstructed *W* color plane, the modified version of the HC method is performed on $I^{RGBW}$ in order to obtain a rough demosaiced image $I^{rough,RGB}$. Secondly, we propose an error compensation-based demosaicking method to reduce the error of the *R*, *G*, and *B* color values in $I^{rough,RGB}$, obtaining the improved demosaiced RGB full-color image $I^{improved,RGB}$. In the third stage, an $I^{RGBW}$-based refinement process is proposed to zeroize the error RGBW CFA image of $I^{improved,RGB}$, achieving a better demosaiced RGB full-color image. Based on the testing RGBW CFA images that were collected from the Kodak and IMAX datasets, the comprehensive experimental data have justified the CPSNR and perceptual effect merits of our method relative to the HC${}^{bicubic}$ and HC${}^{Kiku}$ methods [11], the pansharpening-based method [13], and the deep learning-based method [16].

Our future work is to adjust our demosaicking method to tackle the SNR limitation problem when considering the effect of read noise and limited full well charge of small pixels in more modern sensors.

## Figures and Tables

**Table 1:** CPSNR MERIT of the Proposed Three-Stage Method.

		HC${}^{bicubic}$ [11]	HC${}^{Kiku}$ [11]	VA [12]	Pansharpening-Based Method [13]	Deep Learning-Based Method [16]	Proposed
CPSNR	IMAX	29.32595	31.4849	31.05877	33.26039	**33.98561**	33.47046
	Kodak	28.84192	31.7211	35.95515	33.58083	34.85267	**36.67467**
Average CPSNR		29.08	31.60	33.50	33.42	34.42	**35.072**
Average CPSNR gain		5.988635	3.46957	1.56561	1.65196	0.65343	

**Table 2:** CPSNR, SSIM, and ΔE Merits of the Proposed Three-Stage Method.

		HC${}^{bicubic}$ [11]	HC${}^{Kiku}$ [11]	VA [12]	Proposed
CPSNR	IMAX	29.32595	31.4849	31.05877	**33.47046**
	Kodak	29.26757	31.85273	35.73768	**36.57367**
Average CPSNR		29.29676	31.66882	33.39823	**35.02207**
Average CPSNR gain		5.72531	3.353255	1.623845	
SSIM	IMAX	0.8921	0.915778	0.898775	**0.933092**
	Kodak	0.895423	0.933169	0.973259	**0.975752**
Average SSIM		0.8937615	0.924474	0.936017	**0.954422**
Average SSIM gain		0.0606605	0.029949	0.018405	
ΔE	IMAX	3.531127	2.777121	2.966827	**2.392574**
	Kodak	3.708511	2.598007	**2.122344**	2.173272
Average ΔE		3.619819	2.687564	2.544586	**2.282923**
Average ΔE gain		−1.336896	−0.40464	−0.26166	
